# Case Report: Effects of a simulated jiu-jitsu fight on hematological, biochemical, and electrolyte responses in athletes with transfemoral amputation: a multiple-case study

**DOI:** 10.3389/fspor.2026.1893195

**Published:** 2026-08-07

**Authors:** Jaqueline Lopes, Anibal Monteiro Magalhães Neto, Luis Carlos Gonçalves, Claudia Marlise Balbinotti Andrade

**Affiliations:** Federal University of Mato Grosso, Cuiabá, Brazil

**Keywords:** adapted sport, hematological parameters, rehabilitation, sports medicine, sports physiology

## Abstract

**Background:**

Blood tests are commonly performed in sporting contexts to screen for possible medical conditions associated with physical performance. This study aimed to: i) investigate the effects of jiu-jitsu fighting on cell damage; and ii) monitor acute hematological patterns.

**Methods:**

Blood samples were collected from three middle-aged men, with transfemoral amputation, practitioners of jiu-jitsu parasport at a professional level, before and after a simulated fight, to screen for possible medical conditions that could compromise performance and cause sports injuries. Ethical aspects were respected. The following analyses were performed: blood count, markers of muscle damage, electrolytes, and analysis of iron stores.

**Results:**

Ferritin levels were above reference values for two participants, even before the simulated fight. Urea and creatinine were within reference values. The other hematological variables also increased, but remained within the reference limits considered normal. Electrolyte concentrations (Na, K, Cl −, and Mg) showed significant changes in K and Mg after the simulated fight (*p* < 0.05). Significant increases were also observed for lactate and LDH (*p* < 0.05).

**Conclusions:**

It is concluded that the simulated fight led to alterations in all observed parameters, even though the majority of values remained within limits considered normal.

## Background

The popularity of jiu-jitsu practiced by athletes with disabilities is growing in different parts of the world. Sports practice results in metabolic and physical adaptations in the high-performance athlete's body, as chronic training improves performance and physiological parameters, due to greater intensity and volume, which are specific to training and competition. In turn, intermittent efforts, with dynamic and uninterrupted movements on the ground, in the presence of physical limitations, characterize the jiu-jitsu parasport practiced by amputees (1). In view of the complexity and adaptive demands of this sport, associated with the limitations of physical disability, investigation methods to prevent health disorders are important (2–4).

During the fight, the athlete is subjected to moments of high and low intensity. In moments of high intensity, the body uses the metabolic pathway of phosphocreatine and muscle glycogen, through intramuscular reserves, to synthesize the necessary ATP. However, the use of glycogen for ATP resynthesis results in lactate accumulation. Other biochemical outcomes resulting from high intensity efforts include greater production of free radicals and an abrupt increase in oxygen consumption (5). The change in oxygen consumption is directly related to the production of free radicals.

The presence of free radicals triggers a series of antioxidant defense mechanisms, which aim to regulate intracellular levels to control cellular damage. The imbalance between oxidizing agents and antioxidants, with greater activity of the oxidizing action, generates the process popularly known as oxidative stress. Subsequently, biomolecular oxidation occurs with impairment of several biological functions, in addition to homeostatic imbalance, oxidative damage to cells and tissues, and possible compromise of proteins and DNA (6). However, all these adaptations depend on the intensity of the exercise, where volume and time are fundamental variables for interpreting responses.

Regarding the above, sports performance is related to the adequate distribution of training loads and sufficient recovery intervals (7). Therefore, monitoring hematological and biochemical markers is important as the findings support periodization and prevent the harmful effects of high-intensity training. This is particularly relevant when considering athletes with disabilities, who already present functional limitations as a result of acquired or congenital problems. Furthermore, the sports literature on athletes with disabilities still presents several gaps, requiring more studies on specific sports that are little explored, such as jiu-jitsu.

In this context, protection and concern for the athlete's health is important. In this regard, the study by Ljungqvist et al. (8) mentions that although sports participation at a high-performance level for athletes with disabilities is important, as it assists in social inclusion, and the development of physical skills and functional independence of the subjects, minimizing various effects of a sedentary lifestyle and risk of premature mortality, the risks of musculoskeletal disorders in this group are undeniable, and knowledge of the adaptive effects of sport is essential, so that the health team can adequately manage the athlete, providing conditions for sporting practice at safe levels.

In light of these findings, the present study was based on the question of the need to screen high-performance athletes with disabilities. In this regard, the study by Trojian et al. (9), indicates that the sports team plays a fundamental role in preventing diseases and improving performance, which is possible through the monitoring of imaging and laboratory tests that investigate the functioning of body systems in response to exercise. The author states that testing athletes’ blood in search of abnormalities can sometimes be Effective. For example, testing hemoglobin and ferritin levels has provided promising evidence, backed by robust scientific results. Furthermore, in sports that require strength, testing glucose seems to make sense, while muscle damage markers are important in demonstrating the body's recovery condition. Likewise, electrolytes indicate possible situations of dehydration. All of these impacts are harmful to the athlete's performance and health and therefore, these analyses seem to be indicated.

Therefore, the current study aimed to: i) investigate the effects of jiu-jitsu fighting on cell damage; and ii) monitor acute hematological patterns.

## Materials and methods

This multiple case study was conducted and reported in accordance with the CARE (CAse REport) guidelines for case reports and case series. The recommendations were followed to ensure transparency in participant characterization, data collection procedures, outcome reporting, and ethical aspects.

### Participants

Three middle-aged male athletes, practitioners of jiu-jitsu parasport, with acquired transfemoral amputation participated in the study (Table 1). This is a convenience sample, so the low sample number is justified by the logistical difficulty in grouping a large number of participants with the same delimited characteristics. The three participants each had to have part of their lower limb amputated due to a motorcycle accident. The eligibility criteria adopted included practicing jiu-jitsu parasport for a period of more than 24 months, at a high-performance level. The recruitment period for the study started on 02-02-25 and ended on 05-05-25.

**Table 1:** Participant characteristics.

Characteristic	Participant 1 (P1)	Participant 2 (P2)	Participant 3 (P3)
Age (years)	46	48	43
Stature (m)	1.60	1.66	1.58
Body mass (kg)	85.6	102.5	53.3
Body mass index (kg.m^−2^)	25.8	41.1	21.4
Fat percentage (%)	28.7	55.9	33.8
Training time per week (hours)	10	10	5

Legend: P, participant.

Additionally, to be included, participants were required to report the absence of anemia, infection, diabetes, cardiovascular disease, and musculoskeletal injuries in the six months prior to data collection. The athletes were instructed to abstain from the use of anti-inflammatories, painkillers, alcoholic beverages, and tobacco and not to exercise in the 24 h before the procedures. Participants were requested to follow a diet appropriate to the demands of the fight and they did not receive special supplements.

The anonymity of the participants was guaranteed. Masking of participants, evaluators, and investigators was carried out regarding the results, hypotheses, and outcomes that would be analyzed.

### Clinical and sports history

All participants presented unilateral transfemoral amputation resulting from traumatic traffic accidents. Athletes 1 and 2 had right-sided amputations, whereas Athlete 3 had a left-sided amputation. Time since amputation was 20, 18, and 12 years, respectively. All athletes routinely used lower-limb prostheses during activities of daily living; however, none used prosthetic devices during training sessions or official para-jiu-jitsu competitions.

The athletes exhibited extensive competitive experience in para-jiu-jitsu. Athlete 1 had practiced jiu-jitsu for approximately 20 years, including experience prior to the amputation event, whereas Athletes 2 and 3 reported 15 and 8 years of practice, respectively. All participants had achieved high-level competitive success, including regional, national, and world championship titles, characterizing them as elite para-athletes.

### Ethical aspects

All participants were informed about the procedures and objectives of the study and, after agreeing, signed an Informed Consent Form (TCLE), ensuring their privacy rights. All procedures were previously approved by the Research Ethics Committee involving human beings at the Federal University of Mato Grosso (Araguaia campus, opinion number: 2.997.241).

### Timeline

This is a multiple-case study. Data collection was carried out during the 1st semester/2025 and took place at the participants’ usual training center (Grace Barra Academy) located in the municipality of Barra do Garças (Brazil). Data analysis was performed at the Federal University of Mato Grosso, Cuiabá Campus (Brazil). All procedures were carried out under standardized ambient conditions (temperature: 28 ± 1° C, relative humidity: 84%).

Initially, participants underwent an anthropometric assessment (Table 1) using a scale (Tanita BC554, Iron Man/InnerScaner, Tanita, Illinois, USA) and a stadiometer (Sanny, American Medical do Brasil, São Paulo, Brazil). The body mass index (BMI) was calculated as body mass divided by height squared. Calculations of body mass, height, and BMI were corrected, considering the absence of part of the limb. Skinfolds (pectoral, mid-axillary, tricipital, subscapular, abdominal, suprailiac, and medial thigh) were measured using a Harpenden adipometer (John Bull British Indicators®, England), with a constant pressure of 10 g/mm and an accuracy of 0.2 mm.

**Figure 1:**
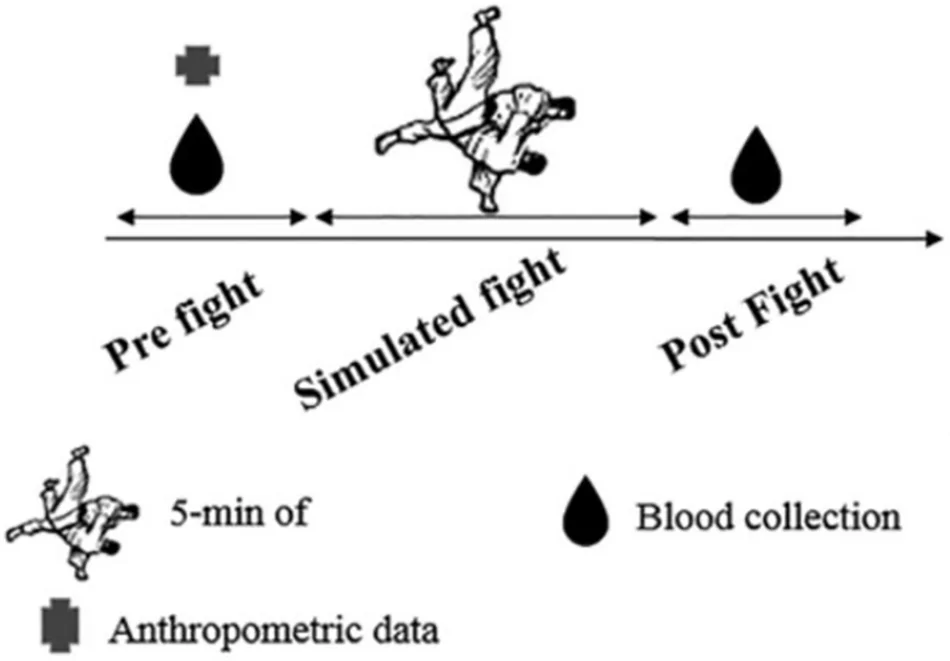
Study design.

Data on hematological and biochemical parameters were determined from blood collected immediately before (pre) and immediately after (post) a simulated fight. Figure 1 illustrates the complete timeline of the study procedures. Given the cross-sectional nature of the investigation, all assessments were conducted within a single data collection session, comprising pre-fight evaluations, the simulated jiu-jitsu match, and immediate post-fight laboratory analyses. Thus, the figure summarizes the entire assessment process and the chronological sequence of data collection performed in the present study.

**Figure 2:**
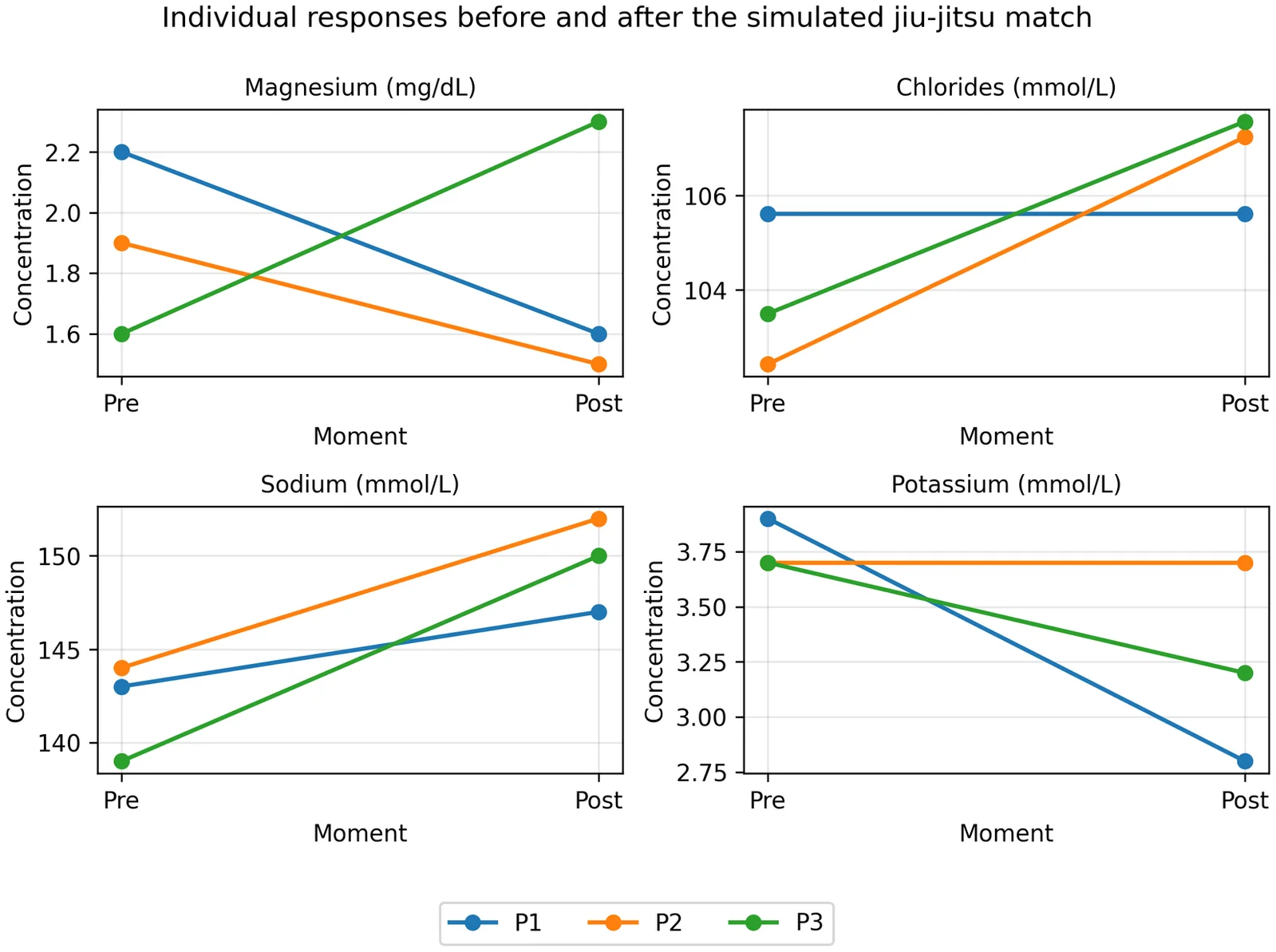
Serum concentrations of magnesium, chlorides, sodium, and potassium before and after the fights in both groups. *Δ*, delta.

### Intervention/exposure

The order of fights between the three participants was defined by prior randomization. A warm-up was performed with light intensity jiu-jitsu movements, characterized by low heart rate and low strength requirements, for five minutes. The simulated fight protocol took place in accordance with the rules of the Brazilian Jiu-Jitsu Sports Confederation, excluding any type of submission before the scheduled time. In these cases, the participants were separated and instructed to return to the fight immediately. Maximum effort and similar activity were recommended for everyone. Participants fought with athletes without disabilities, not included in the study, who were previously trained and oriented regarding standardized fighting behavior, in order to minimize the influence of possible biases (10).

### Outcomes

#### Blood sampling and analysis

Two samples containing 5 mL of venous blood were extracted from the participants’ antecubital vein, one immediately before and the other immediately after the simulated fight. Vacutainer tubes containing ethylenediaminetetraacetic acid (EDTA) with anticoagulant were used. After collection, the blood samples were centrifuged at 2,500 rotations per minute, for a period of 10 min, at room temperature, in order to isolate the serum. Afterwards, the samples were coagulated for 30 min. The serum was aliquoted in an Eppendorf tube and stored at an average temperature of – 80 °C until the biochemical analyses.

#### Assessment of hematological and biochemical parameters

The hematological parameters analyzed were: red blood cells, hematocrit, and hemoglobin measured using an analyzer (ABX Pentra 60 HORIBA). Urea, creatinine, uric acid, glutamic-oxaloacetic transaminase (TGO), glutamic-pyruvic transaminase (GPT), albumin, lactate dehydrogenase (LDH), glucose and electrolytes (sodium (Na), potassium (K), chloride (Cl −), and magnesium (Mg) were determined by spectrophotometric techniques.

#### Data presentation

Due to the low sample number that would limit the statistical power for group analyses and to facilitate data visualization and comparison, absolute values for each participant are presented. The changes (chang %) in the variables analyzed between the pre- and post-fight moments were calculated by averaging the percentage differences (10). Statistical analysis was performed using a paired Student's t-test to compare the variables measured before and after the simulated combat training session. A two-tailed test was applied, and statistical significance was set at *p* < 0.05.

## Results

The results of the hematological parameters obtained before and after the fight are presented in Table 2, Figure 2. It can be noted that only the values of lymphocytes, total leukocytes, and neutrophils were above normal reference values after the simulated fight. The other hematological variables, despite increasing, remained within the reference limits considered normal.

**Table 2:** Hematological parameters.

Parameter	P1	P2	P3	Mean	SD	Δ%	*p*-value
*Hematocrit (%) - RV: 40 to 50%*							
*Pre*	42.5	47.1	40.5	43.3	2.4	2.4	0,026*
*Post*	45.8	51.8	43.2	46.9	3.2	3.2	
*Δ%*	7.76	9.97	6.66	8.13	1.2	1.2	
*Red blood cells (millions/mm3) – RV: 4.3–5.8*							
*Pre*	5.51	5.66	4.96	5.37	0.2	0.2	0,016*
*Post*	5.79	6.04	5.21	5.68	0.3	0.3	
*Δ%*	5.08	6.71	5.04	5.61	0.73	0.73	
*Hemoglobin (g/dL) – RV: 13.0–16.9*							
*Pre*	15.7	17.7	15.4	16.6	0.9	0.9	0,008*
*Post*	16.6	18.8	16.2	17.2	1.06	1.06	
*Δ%*	5.73	6.21	5.19	5.71	0.34	0.34	
*Platelets (unit mm3) – RV: 150,000 to 400,000*							
*Pre*	240,000	204,000	201,000	215,000	16,666,66	16,666,66	0,06
*Post*	276,000	237,000	275,000	262,667	17,111,11	17,111,11	
*Δ%*	15	16.1	36.8	23	9.4	9.4	
*Total leukocytes (unit mm3) – RV: 4,000 to 11,000*							
*Pre*	7,590	8,970	7,430	7,997	648,8	648,8	0,01*
*Post*	11,650	13,320	13,540	12,837	791,11	791,11	
*Δ%*	53.49	48.49	82.23	61	13,88	13,88	
*Neutrophils (unit mm3) – RV: 1.500 a 8.000 celules/mm^3^*							
*Pre*	3,719	4,574	4,755	4,349	420,22	420,22	0,02*
*Post*	5,592	5,994	7,311	6,299	674,66	674,66	
*Δ%*	50.36	31.04	53.75	45	9,33	9,33	
*Lymphocytes (mm3 unit) – RV: 900 to 4,000*							
*Pre*	3,263	3,588	1,931	2,927	664,22	664,22	0,01*
*Post*	5,475	6,526	5,145	5,715	540,44	540,44	
*Δ%*	67.79	81.88	166.44	105	40.71	40.71	

Legend: P, participant; RV, reference values; SD, standard deviation.

* statistical significance *p* < 0.05.

The biochemical parameters are shown in Table 3. Significant increases were observed for lactate and LDH after the fight (*p* < 0.05). Ferritin was normal for participant 3, and was elevated even before the simulated fight for participants 1 and 2. TGO and TGP enzymes increased after the fight in all participants, but were within normal reference values. Urea increased in participant 2, decreased in participant 1, and did not change in participant 3. Creatinine and albumin increased in all participants, but within normal reference values. Uric acid decreased in all participants, but within normal reference values. Glucose increased in participants 1 and 3, but decreased in participant 2.

**Table 3:** Biochemical parameters.

Parameter	P1	P2	P3
*Lactate (mmol/L) – RV: 1.0 to 4.0*			
*Pre*	2.8	1.4	1.4
*Post*	15.7	16.4	16.8
*Δ%*	460.7	1,073.5	1,100
*LDH (U/L) – RV: 100–190*			
*Pre*	355	360	318
*Post*	401	508	372
*Δ%*	12.95	41.11	16.98
*Ferritin (ng/mL) – RV: 23 to 336*			
*Pre*	379	920	186
*Post*	406	1,030	223
*Δ%*	7.12	11.95	19.89
*Glutamic oxaloacetic transaminase (U/L) – RV: 5–40*			
Pre	29	34	36
Post	34	47	41
*Δ%*	17.24	38.23	13.88
*Glutamic-pyruvic transaminase (U/L) – RV: 7–56*			
*Pre*	50	29	27
*Post*	57	34	28
*Δ%*	14	17.24	3.70
*Urea (mg/DL) – RV: 13 to 43*			
*Pre*	24	23	17
*Post*	21	26	17
*Δ%*	−12.5	13.05	0
*Creatinine (mg/dL) – RV: 0.7 to 1.3*			
*Pre*	0.54	0.6	0.5
*Post*	0.7	0.8	0.6
*Δ%*	29.62	33.3	20
*Uric Acid (mg/dL) – RV: 3.4–7.0*			
*Pre*	6.04	5.28	6.61
*Post*	5.6	4.96	6.52
*Δ%*	−7.28	−6.06	−1.36
*Albumin (g/L) – 30–300*			
*Pre*	43	42	41
*Post*	49	47	48
*Δ%*	13.95	11.90	17.07
*Glucose (mg/dL) – until 140*			
*Pre*	110	135	78
*Post*	171	90	93
*Δ%*	55.45	−33.33	19.23

Legend: P, participant; RV, reference values.

There were no significant changes in the pre and post-fight values for chloride (Cl) and sodium (Na), while significant changes were observed in the values of magnesium (Mg) and potassium (K) (*p* < 0.05).

## Discussion

The main objective of this study was to measure the effects of a jiu-jitsu fight (simulated competition) on acute hematological and biochemical parameters in high-performance amputee athletes. No previous studies were identified exclusively including this participant profile. Jiu-jitsu is a predominantly anaerobic activity, which requires physical conditioning to avoid fatigue and promote good recovery. Overall, all markers increased after the simulated fight. Thus, the results showed that the simulated fight changed most of the analyzed parameters, mainly the biochemical markers, although the majority also remained within reference values considered normal.

Our findings are partially consistent with previous observations in elite para-jiu-jitsu athletes, in whom acute exercise induced significant biochemical and hematological fluctuations associated with high-intensity combat efforts (2–4).

Total leukocyte and neutrophil values were elevated after the fight in all three participants. This change corroborates the scenario of acute inflammation triggered in response to exercise. Lee et al. (11) mention that the change in the total number of leukocytes and neutrophils after exercise can be induced by stress hormones, such as epinephrine, norepinephrine, and cortisol, indicating a promising recovery scenario if hormonal modulation is controlled. The increase in leukocyte, neutrophil, and lymphocyte counts is consistent with exercise-induced leukocytosis. High-intensity exercise promotes sympathetic activation and the release of catecholamines and cortisol, leading to the mobilization of immune cells from marginal pools into the circulation. This response represents a physiological adaptation rather than a pathological inflammatory process and is commonly observed after strenuous exercise. Furthermore, mechanical stress and muscle microdamage may stimulate cytokine production, contributing to transient immune cell trafficking (11).

Regarding lactate, significant increases were observed for the three participants (460.7%, 1,073.5%, 1,100%, respectively). Previous studies also observed increases in lactate in jiu-jitsu athletes with disabilities (1, 10, 12). Thus, marked increase in lactate concentrations reflects a high contribution of anaerobic glycolysis during the simulated fight. Jiu-jitsu involves repeated bouts of isometric contractions, explosive movements, gripping actions, and short recovery periods, which increase ATP demand beyond the capacity of oxidative metabolism. Consequently, pyruvate production exceeds mitochondrial oxidation rates, resulting in lactate accumulation. The particularly elevated values observed may also reflect the high competitive level of the athletes and the intense metabolic requirements of para-jiu-jitsu.

The LDH values after the fight increased significantly only in participant 2. Interestingly, participant 2 had the greatest overweight and body fat level, with such data, this being directly associated with the increase in LDH. This is because obesity represents a chronic disease that compromises the proper functioning of the enzyme in terms of its ability to break down glucose. There were more discrete increases for the other participants. It is known that exercise-induced muscle damage is associated with the performance of repetitive eccentric exercises at high intensity. Therefore, the perception of effort and the different level of conditioning between participants may justify the differences in results between them (13). Consequently, the damage can result in impairments in force production, range of motion, and greater perception of delayed muscle soreness. Therefore, the implementation of recovery strategies in this profile seems to be pertinent. Furthermore, increased LDH activity may indicate disruption of muscle cell membrane integrity caused by mechanical stress during combat. Because LDH is an intracellular enzyme, its release into circulation is often associated with transient muscle membrane permeability changes and exercise-induced muscle damage. The moderate elevations observed suggest physiological tissue stress rather than clinically relevant injury.

An additional finding that deserves consideration is that LDH concentrations were above the reference range in all participants before the simulated match. This observation suggests that the elevated values cannot be attributed exclusively to the acute effects of the combat. In high-performance athletes, increased resting LDH concentrations may reflect chronic training adaptations, repeated exposure to strenuous exercise, ongoing muscle tissue remodeling, and residual muscle damage associated with regular training loads. Therefore, the baseline elevations observed in the present study should be interpreted cautiously, as they likely represent pre-existing physiological characteristics rather than solely exercise-induced responses (14).

Ferritin is not only a marker of iron stores but also an acute-phase protein. Therefore, elevated concentrations may reflect inflammatory activity, repeated training loads, or physiological adaptations associated with chronic exposure to exercise. The elevated baseline values observed in two participants may indicate long-term training adaptations rather than abnormalities in iron metabolism. Ferritin levels were high in two participants (P1 and P2) and within reference values for one participant (P3). Contradictorily, the study by Roy et al. (15), mentions that iron deficiency is a common phenomenon in high-performance sports and can lead to compromised physical performance, with the prevalence of deficiency being higher in adolescents and women. In this regard, these results associated with the behavior of the other variables analyzed lead us to believe that a simulated jiu-jitsu fight, practiced by individuals with disabilities, represents a type of effort that does not seem to be sufficient to result in considerable depletion of fundamental elements of the organism, such as ferritin (1).

It is important to note that elevated ferritin concentrations were already present in two participants before the simulated match, indicating that these findings cannot be attributed exclusively to the acute effects of exercise. Ferritin is influenced by several physiological and clinical factors beyond iron stores, including chronic low-grade inflammation, obesity, metabolic dysfunction, and liver-related conditions. In particular, Participant 2 presented severe obesity, which may have contributed to the elevated ferritin concentrations observed at baseline. Therefore, caution is warranted when interpreting ferritin responses in the present study, as pre-existing individual characteristics may have influenced the observed values.

Creatinine concentrations increased in all participants, while uric acid decreased in all participants. Creatinine and uric acid are associated with increased strength and muscle mass, and previous studies have reported increases in these markers after strength training. Furthermore, uric acid constitutes a metabolic product that is associated with the antioxidant property attributed to its ability to react to biological oxidants (4). Therefore, an increase in this marker indicates a mechanism triggered in response to the production of reactive oxygen species, common during exercise. However, the decrease observed in all participants in the current study indicates that the intensity levels required during jiu-jitsu practice for amputee athletes are possibly insufficient for significant strength demands.

Transaminases (TGO and TGP) increased after the fight, but remained within normal reference values. These markers are indicated to assess liver status, but are also present in other organs, such as muscles, heart, kidneys, red blood cells, and the small intestine. Furthermore, the literature mentions that when considering high-performance athletes, increases after exercise are more associated with muscle damage than possible liver damage.

Analysis of electrolyte concentrations (Na, K, Cl −, and Mg) showed significant changes in K and Mg after the simulated fight (*p* < 0.05). It is possible that the intensity of the fight, the perception of effort, dehydration, and distribution of electrolytes explain the changes observed. This is because sweat is made up of electrolytes that include Na, Cl -, and K, justifying the decrease in these markers after exercise with severe sweating. This factor is very variable between individuals, as highlighted by Lott (16), which explains the possible differences between participants. Lombardo et al. (17) state that large losses of electrolytes can compromise performance and cause sports injuries, which is why adequate monitoring is important. Therefore, these data suggest that depending on the intensity of exercise, fluid/electrolyte intake strategies may be necessary. Alterations in potassium and magnesium may be associated with exercise-induced shifts between intracellular and extracellular compartments. Potassium is released from contracting skeletal muscle fibers during repeated depolarization-repolarization cycles, whereas magnesium participates in ATP-dependent reactions and muscle contraction processes. Changes in both electrolytes may therefore reflect the metabolic demands imposed by the fight and fluid redistribution during exercise (16).

The data presented describe the metabolic responses of amputee athletes after a simulated jiu-jitsu fight, to verify that their health status. The findings are expected to promote timely knowledge to support the proposition of preventive strategies, periodization training to optimize performance, and prescription of protocols to reduce the risk of injuries associated with sports practice. The data make it possible to map the behavior of biochemical and hematological markers after exercise, specifically for amputee athletes practicing high-performance jiu-jitsu.

Perhaps the biggest limitation of this study is the lack of previous data presented exclusively on amputee athletes, which made comparisons and deeper discussions about the findings impossible. Regarding the reduced number of the sample, it is believed that case studies, including participants with rare and still little-researched characteristics, are important for producing knowledge and discovering unknown parameters. This information is important to support future investigations and the proposal of strategies regarding athlete management. In this regard, the study by Halperin (18) explains that case studies are advantageous when considering the use of data as a strategy to support practical sport contexts, in addition to helping scientists and sport professionals with common interests to connect and produce future collaborations for the advancement of science in a given area of knowledge.

Finally, broad knowledge of the athlete's physiological conditions makes it possible to propose the periodization and prescription of recovery protocols individualized to the needs of each sporting modality. We must remember that high-performance athletes often present higher risk exposure than people in general and, therefore, screening tests constitute important preventive and health monitoring measures to be carried out from time to time (12, 18, 19).

The present study has some limitations that should be considered when interpreting the findings. An important limitation of the present study is the very small sample size, which restricts statistical power and limits the applicability of inferential analyses. Consequently, statistically significant findings should be interpreted with caution and considered exploratory, serving primarily as preliminary evidence to support future studies in para-sport populations. The small number of participants and the case-series design limit the generalizability of the results to other para-athlete populations. Furthermore, only the acute responses to a single simulated jiu-jitsu match were evaluated, without longitudinal follow-up. Despite these limitations, the study provides novel information regarding hematological, biochemical, and electrolyte responses in elite para-jiu-jitsu athletes with transfemoral amputation, a population that remains substantially underrepresented in the scientific literature. These findings may contribute to the development of future investigations and support periodic health monitoring strategies in para-sport settings.

## Conclusions

In conclusion, a simulated jiu-jitsu match elicited acute hematological, biochemical, and electrolyte changes in elite athletes with transfemoral amputation. Although the findings are limited by the small sample size and exploratory design, they provide preliminary evidence regarding the physiological responses of a population that remains underrepresented in the scientific literature. These observations may contribute to future investigations and support the development of monitoring strategies for athletes participating in para-sport environments.

## Patient perspective

The participants have been involved in our research and monitoring program since 2019 and have undergone regular assessments of physical, functional, and metabolic variables. Throughout this period, they have consistently reported confidence in the feedback, preventive health guidance, and support provided by the research team. In addition to research-related evaluations, the athletes receive ongoing theoretical and practical support regarding training, health management, and injury prevention. When musculoskeletal complaints or injuries occur, appropriate professional assistance and follow-up are also provided. Although a formal patient perspective interview was not conducted for publication purposes, the participants have maintained a positive and collaborative relationship with the research team throughout the monitoring process.

## Clinical relevance

- Sports are an opportune strategy for the rehabilitation and integration of individuals with disabilities;
- Blood tests are useful for tracking health conditions associated with performance.
- In amputees, hematological and biochemical parameters increase after a simulated fight, but significant increases above reference values were observed for ferritin, magnesium and potassium
- It is believed that the data presented are useful for describing previously unknown behaviors, being important for prescribing individualized conduct in the sports management of this athlete profile that has such particular characteristics.

## Data Availability

The raw data supporting the conclusions of this article will be made available by the authors, without undue reservation.
